# Effect of Temperature on Growth of *Vibrio paraphemolyticus* and *Vibrio vulnificus* in Flounder, Salmon Sashimi and Oyster Meat 

**DOI:** 10.3390/ijerph9124662

**Published:** 2012-12-13

**Authors:** Yoo Won Kim, Soon Ho Lee, In Gun Hwang, Ki Sun Yoon

**Affiliations:** 1 Department of Food and Nutrition, Hoeki-dong Dongdaemun-Ku, Kyung Hee University, Seoul 130-701, Korea; E-Mail: ryuwon@gmail.com; 2 Food Microbiology Division, National Institute of Food and Drug Safety Evaluation, Korea Food and Drug Administration, Chungcheongbuk-do 363-700, Korea; E-Mails: leesh13@kfda.go.kr (S.H.L.); hwangig@kfda.go.kr (I.G.H)

**Keywords:** *V. parahaemolyticus*, *V. vulnificus*, flounder, salmon, oyster, growth kinetics

## Abstract

*Vibrio parahaemolyticus* and *Vibrio vulnificus* are the major pathogenic *Vibrio* species which contaminate ready-to-eat seafood. The purpose of this study was to evaluate the risk of human illness resulting from consumption of ready-to-eat seafood such as sashimi and raw oyster meat due to the presence of *V. parahaemolyticus* and *V. vulnificus.* We compared the growth kinetics of *V. parahaemolyticus* and *V. vulnificus* strains in broth and ready-to-eat seafood, including flounder and salmon sashimi, as a function of temperature. The growth kinetics of naturally occurring *V. vulnificus* in raw oyster meat was also evaluated. The minimum growth temperatures of *V. parahaemolyticus* and *V. vulnificus* in broth were 13 °C and 11 °C, respectively. Overall, significant differences in lag time (LT) and specific growth rate (SGR) values between flounder and salmon sashimi were observed at temperatures ranging from 13 °C to 30 °C (*p* < 0.05). The growth of naturally occurring *V. vulnificus* reached stationary phase at ~4 log CFU/g in oysters, regardless of the storage temperature. This data indicates that the population of *V. vulnificus* in oysters did not reach the maximum population density as observed in the broth, where growth of *V. vulnificus* and *V. parahaemolyticus* isolated from oysters grew up to >8 log CFU/mL.

## 1. Introduction

*Vibrio parahaemolyticus* and *Vibrio vulnificus* are the most common *Vibrio* species associated with illnesses resulting from consumption of raw or partially cooked seafood worldwide [1,2,3,4]. In comparison to *V. parahaemolyticus, V. vulnificus* is known to be more dangerous because it causes septicemia in immunocompromised individuals, and *V. vulnificus* septicemia is associated with a greater than 50% mortality [5]. It was reported that infections caused by pathogens commonly transmitted through food have declined, or are approaching targeted national levels, with the exception of *Vibrio* infections [6]. In Asia, the number of reported *V. parahaemolyticus* infections has increased significantly [7]. The highest incidence of outbreaks (33) and illness (634) due to *V. parahaemolyticus* were reported between 2006 and 2010, which accounted for 17.1% (range from 13.9% to 20%) of all foodborne illnesses caused by bacterial pathogens in Korea [8].

Flounder (Gwang-eo, *Paralichthys* spp.) and salmon (*Salmonidae* spp.) are the most frequently fish consumed as sashimi (raw fish) and sushi (a roll of cold rice with fish) in Asia [9]. Several cases of food poisoning due to the consumption of salmon sashimi contaminated with *V. parahaemolyticus* have been reported. Thus, it is important to control the growth of *V. parahaemolyticus* and *V. vulnificus* in sashimi and sushi, which are also becoming more popular in many other countries [10]. In addition, oysters are also frequently eaten raw and are the food most commonly associated with *Vibrio* infection in many countries [11,12]. Due to the ubiquitous nature of *Vibrio* species in marine environments, it is impossible to obtain seafood free of these bacteria. Therefore, it is necessary to limit the growth of *Vibrio* spp. in contaminated seafood in order to minimize the risk of foodborne illnesses due to seafood consumption. Unfortunately, refrigeration and freezing cannot completely destroy *V. parahaemolyticus* [13] and temperature abuse as well as mishandling of seafood at retail markets and food-service establishments may cause the rapid growth of *V. parahaemolyticus* and *V. vulnificus* [14,15]. Thus, it is important to understand the behavior of *Vibrio* spp. in raw and ready-to-eat seafood under temperature abuse conditions common during distribution and storage at retail markets. 

Burnham *et al.* reported the differing growth and survivability of eight different *V. parahaemolyticus* and *V. vulnificus* strains in broth at a refrigeration temperature of less than 10 °C [16]. Yang *et al.* reported the growth and survival curves for a strain of pandemic *V. parahaemolyticus* inoculated on salmon meat over a temperature range from 0 °C to 35 °C [17]. A few works regarding modeling of the effect of temperature on the growth of *Vibrio.* spp were also reported [18,19,20,21]. Miles *et al.* [18] modeled the effects of temperature and water activity on the growth rate of *V. parahaemolyticus* in broth. Yoon *et al.* [19] developed growth models for pathogenic and nonpathogenic *V. parahaemolyticus* in both broth and oyster slurry. Most recently, the effect of storage temperature (ranging from 3.6 °C to 30.4 °C) on *V. paraemolyticus* viability and counts of total viable bacteria in Pacific oysters was reported [20]. A predictive growth model of *V. vulnicus* in postharvest shellstock oysters were also developed and reported [21]. However, the behavior of *Vibrio* spp. in sashimi as a function of temperature and kind of fish has not been compared. Therefore, the primary objective of this study was to compare the growth kinetics of *V. parahaemolyticus* in flounder and salmon sashimi as a function of temperature, which were also compared to the growth kinetics of *V. vulnificus* and *V. parahaemolyticus* in broth. In addition, growth kinetics of naturally occurring *V. vulnificus* in oyster was evaluated in order to assess the risk involved with the consumption of raw oyster meat if oysters are not promptly refrigerated. 

## 2. Experimental Section

### 2.1. Bacteria Strains

Three strains of *V. parahaemolyticus* (ATCC 17802, 27969, and 33844) and *V. vulnificus* ATCC 27562 were used. All strains were maintained at −70 °C at a concentration of 9.5–10.0 log CFU/mL in nutrient broth (NB; Becton Dickinson, Sparks, MD, USA) with 3% salt that contained 20% glycerol (Sigma Chemical Company, St. Louis, MO, USA). For each experiment, 10 μL of thawed, stock culture of *V. parahaemolyticus* and *V. vulnificus* was inoculated into a 25 mL Erlenmeyer flask that contained 9 mL of sterile NB with 2% salt and tryptic soy broth (TSB) containing 2% salt, respectively. The flasks were then sealed with a foam plug and incubated at 36 °C for 24 h at 140 rpm on a rotary shaker (VS-8480SR, Vision Scientific, Daejun, Korea). One mL of the starter culture was then transferred into 9 mL of phosphate-buffered saline (PBS at pH = 7.4) containing 2.5% salt, which was then serially diluted prior to inoculation into both the broth and sashimi samples. 

### 2.2. Preparation of Inoculation in Broth and Sashimi Samples

To compare the growth kinetics of *V. parahaemolyticus* and *V. vulnificus* in broth, 10 μL of the diluted starter culture of *V. parahaemolyticus* ATCC 33844 (the fastest growing *V. parahaemolyticus* strain among all the strains tested in this study) and *V. vulnificus* ATCC 27562 were inoculated into 9 mL of NB containing 2% salt and TSB containing 2% salt using a sterile pipette in order to give a target population of approximately 2.5–3.5 log CFU/mL, respectively. They were then immediately incubated at 11, 13, 18, 24, 30, and 36 °C without agitation. At selected times post-inoculation, which were determined based on the incubation temperature of the broth, 100 μL aliquots were diluted as appropriate, plated onto thiosulfate-citrate-bile salts-sucrose (TCBS; Difco, Becton Dickinson) agar in duplicate, and incubated aerobically at 36 °C for 18–20 h. The colonies on the TCBS plates were then counted, after which, the counts from the duplicated plates were converted to log numbers. In order to compare the growth kinetics of *V. parahaemolyticus* in flounder and salmon sashimi, fresh flounder and salmon sashimi packs (150 g *per* pack) were purchased from a local grocery market in Seoul, Korea (*Vibrio* spp*.* were not detected in any sashimi samples used in this study). Each sample was aseptically prepared by cutting sashimi into 10 g pieces using the blade of a sterile knife and placed in a sterile Petri dish. Each thin (0.2–0.3 mm) slice of sashimi was then uniformly surface inoculated with 10 μL of the diluted starter culture of *V. parahaemolyticus* using a sterile pipette to give a final concentration of ~3 log CFU/g. The samples were then placed into a stomacher bag, sealed, and stored at 13, 18, 24, 30, and 36 °C. The minimum growth temperature of *V. parahaemolyticus* has been determined from the growth data in broth. At selected times after inoculation, sashimi samples were homogenized (Stomacher, Interscience, Paris, France) for 2 min in 90 mL of sterilized PBS containing 2.5% salt (pH = 7.4). A 1 mL aliquot of diluted homogenate was spread onto TCBS in duplicate and incubated at 36 °C for 24 h. The colonies from the duplicated plates were counted and the counts were converted into log numbers. Each experiment was repeated twice with two replicates *per* experiment. 

### 2.3. Isolation of Natural V. parahaemolyticus and V. vulnificus in Shucked Oysters

Shucked oyster (*Crassostrea gigas*) packs (150 g per pack) cultivated on the Southern coast of Korea were purchased from grocery markets in Seoul. The shucked oyster packs were packed in an insulated container with dry ice and transported to the laboratory within 1 h. One pack was used for each temperature. A microbiological analysis was conducted immediately after arrival. Approximately 25 g of oyster meat (2–3 oysters) was added to 225 mL of phosphate buffered saline (PBS at pH = 7.4) with 2.5% salt, and the mixture was blended in a pulsifier (Microben, Bioproduct LTD, Surrey, United Kingdom) for 30 s. The resulting homogenate was streaked onto TCBS agar and incubated for 24 h at 36 °C. All green and yellow colonies that formed on TCBS agar wer**e** enumerated for total *Vibrios*. The green colonies on TCBS agar were transferred onto Chrome Vibrio agar (CV, Chromagar, Paris, France). The colonies of *V. parahaemolyticus* and *V. vulnificus* were identified as having mauve and blue colors in CV agar, respectively, and were tested again for oxidase and Gram staining. Suspicious oxidase positive and Gram-negative colonies were again confirmed as *V. parahaemolyticus* and *V. vulnificus* using an API 20NE diagnostic strip (Biomerieux, Marcy L’Etoile, France). Each isolated *V. parahaemolyticus* and *V. vulnificus* strain was maintained at −70 °C in NB with 2% salt and TSB containing 2% salt, respectively. 

### 2.4. Determination of Growth Kinetics of V. vulnificus in Shucked Oyster Meat

From September to March in 2010–2011, only *V. vulnifius* was consistently detected in shucked oysters obtained from seafood markets. Nor *V. parahaemolyticus* and *V. vulnificus* were detected at all samples from December to February in 2010–2011. Thus, only the growth kinetics of natural *V. vulnificus* in oyster meat was evaluated in this study. Shucked oyster meat (25 g portions) purchased from a grocery market were placed in a stomacher bag using sterilized tweezers and sealed (under micro-aerobic conditions). Each sample was stored at temperatures of 16, 18, 24, 30, and 36 °C for the growth of naturally occurring *V. vulnificus* in oyster meat. At selected times during storage, samples were diluted into 75 mL with phosphate buffered saline (PBS) with 2.5% salt and homogenized in a pulsifier (Microben) for 30 seconds. The homogenates were serially diluted in sterile PBS with 2.5% salt solution and spread onto Chrome Vibrio (CV) agar, in duplicate and incubated 37 °C for 24 h. The colonies of *V. vulnificus* from duplicated plates were counted and the counts were converted into log numbers. 

### 2.5. Effect of Temperature on Growth Kinetics of V. parahaemolyticus and V. vulnificus.

Growth kinetics of *V. parahaemolyticus* and *V. vulnificus* in broth, sashimi and oyster meat were compared by the construction of growth curves. The growth kinetics parameters including lag time (LT) and specific growth rate (SGR) at each temperature were determined by the modified Gompertz equation using GraphPad Prism 4.0 (GraphPad Software, San Diego, CA, USA) [19]. The equation used was as follows:

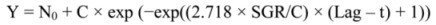
(1)


In this equation, Y is the viable cell count (log CFU/g), N_0 _is the initial log number of cells, C is the difference between the initial and final cell numbers, SGR is the maximum specific growth rate (log CFU/h), LT is the lag time before growth and t is the sampling time. 

For the effect of temperature on the values of LT and SGR, the Davey equation [22] and square root equation [23] were used, respectively. The Davey equation for LT was used as follows:

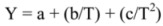
(2)
where Y is LT (day), a, b, and c are regression coefficients without biological meaning, and T is temperature. The square-root equation for SGR was used as follows:

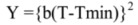
(3)
where Y is the growth rate, *b* is the regression coefficient, and Tmin is the theoretical minimum temperature required for the growth of the organism to occur. The goodness-of-fit of the data was evaluated based on the coefficient of determination (R^2^), which was provided by GraphPad Prism. 

### 2.6. Statistical Analysis

The experiment was repeated twice with two replicates per experiment. The obtained results were analyzed using the Statistical Analysis System, SAS V 9.1 (SAS Institute Inc., Cary, NC, USA). The significant differences among the groups were determined by analysis of variance (ANOVA) and the means were also separated using t-tests (*p* < 0.05). 

## 3. Results and Discussion

### 3.1. Comparison of Growth Kinetics of V. parahaemolyticus and V. vulnificus in Broth

In this study, we used the fastest growing *V. parahaemolyticus* strain 33844 among all the strains tested in this study (data not shown) and compared it to the growth kinetics for *V. vulnificus* strain 27562 in broth at 11, 13 18, 24, 30, and 36 °C (Table 1). The growth curves in broth were fitted well to a Gompertz equation with a high degree regarding goodness-of-fit (R^2^ = 0.995 to 997). Lag time (LT), specific growth rate (SGR), and maximum population density (MPD) were compared between *V. parahaemolyticus* and *V. vulnificus* as a function of temperature. Significant differences (*p* < 0.05) in the values of LT, SGR and MPD between *V. parahaemolyticus* and *V. vulnificus* were also observed at all temperatures. In addition, higher SGR of *V. vulnificus* was observed in comparison to that of *V. parahaemolyticus.* The average MPD values of *V. parahaemolyticus* and *V. vulnificus* were 9.37 and 8.90, respectively. Although *V. parahaemolyticus* strain 33844 (purchased from ATCC) was the fastest growing strain among the tested strains in our study, its growth was much slower than that of the pathogenic strain JL 223 isolated from 72 Korean raw oysters used in a previous study [19]. At 20 °C, a longer LT value (10.32 h) for *V. parahaemolyticus* strain 33844 in broth was predicted in this study, compared to LT values for pathogenic (8.16 h) and nonpathogenic (6.25 h) *V. parahaemolyticus* isolated from raw oysters. 

**Table 1:** Effect of temperature on Lag time (LT) and specific growth rate (SGR), and maximum population density (MPD) of *V. parahaemolyticus* and *V. vulnificus* in broth.

	Strain	11 °C	13 °C	18 °C	24 °C	30 °C	36 °C
LT (h)	*V. parahaemolyticus*	-	48.64 ± 5.23 ^A^	16.94 ± 2.33 ^B^	2.52 ± 0.16 ^C^	2.22 ± 0.33 ^C^	1.61 ± 0.87 ^C.D^
	*V. vulnificus*	127.80 ± 4.97 ^A^	-	10.49 ± 3.17 ^B^	4.60 ± 1.09 ^C^	2.40 ± 0.72 ^D^	1.46 ± 0.23 ^E^
SGR (log/h)	*V. parahaemolyticus*	-	0.035 ± 0.04 ^E^	0.160 ± 0.14 ^D^	0.561 ± 0.02 ^C^	0.847 ± 0.04 ^B^	1.184 ± 0.35 ^A^
	*V. vulnificus*	0.027 ± 0.08 ^D^	-	0.365 ± 0.21 ^B^	0.687 ± 0.22 ^C^	1.029 ± 0.31 ^A^	1.096 ± 0.28 ^A^
MPD (log)	*V. parahaemolyticus*		9.48 ± 0.11 ^A^	9.66 ± 0.25 ^A^	9.12 ± 0.23 ^A.B^	8.86 ± 1.03 ^B^	9.74 ± 0.62 ^A^
	*V. vulnificus*	7.54 ± 1.34 ^C^	-	8.69 ± 0.77 ^B^	9.66 ± 0.29 ^A^	9.62 ± 0.32 ^A^	9.00 ± 0.85 ^A,B^

Capital letters (A, B, C, D, E) mean (n = 4) within a row with different superscripts are significantly different at the *p* < 0.05 level.

Compared to the SGR values of pathogenic (0.42 log CFU/h) and nonpathogenic *V. parahaemolyticus* (0.42 log CFU/mL), a lower SGR value (0.30 log CFU/h) was also predicted for *V. parahaemolyticus* strain 33844. This indicates that care must be given when selecting a strain to develop a growth predictive model, and various strains isolated from seafood must be compared for their growth kinetics under various environmental conditions. These results again confirm the results regarding the study conducted by Burnham *et al.* [16], where strain to strain differences in the growth and survival of various *V. parahaemolyticus* and *V. vurnificus* were observed at refrigeration temperatures. 

The effect of temperature on the values of LT and SGR were determined and modeled (Table 2). The Davey equation for LT and the square root equation for SGR were used for the data collected in the broth. The minimum growth temperatures of *V. parahaemolyticus* and *V. vulnificus* in broth were 13 °C and 11 °C, respectively. In contrast to the results of this study, the minimum growth temperature of *V. parahemolyticus* in broth has been reported to be 5 °C and 8.3 °C [18]. Yoon *et al.* [19] also reported that pathogenic and nonpathogenic *V. parahaemolyticus* grew at 10 °C in broth. Burnham *et al.* [16] observed that five of eight *V. parahaemolyticus* strains and seven of eight *V. vulnificus* strains had increases in viable counts during 10 days of storage at 8 °C and 10 °C, respectively, with significant differences in growth between the various strains. 

### 3.2. Comparison of Growth Kinetics of V. parahaemolyticus in Flounder and Salmon Sashimi

**Figure 1:**
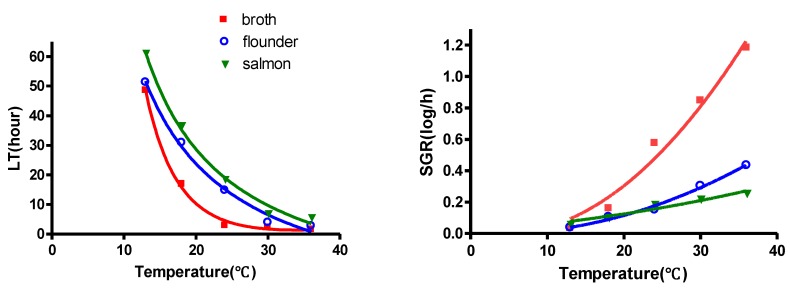
Effect of temperature on the values of Lag time (LT) and Specific growth rate (SGR) of *V. parahaemolyticus* in broth, flounder, and salmon sashimi. Data (n = 4) for LT and SGR were fitted using Equations (2) and (3), respectively.

The results of the present monitoring study showed that no contamination of *V. parahaemolyticus* was found in flounder and salmon sashimi from three different grocery markets in Korea. The growth curves for *V. parahaemolyticus* in flounder and salmon sashimi also fitted well the modified Gompertz equation, with a high degree of goodness-of-fit (R^2 ^= 0.9394 to 0.9948). The values of LT, SGR, and MPD were compared between flounder and salmon sashimi (Table 3). The minimum growth temperature of *V. parahaemolyticus* in flounder and salmon sashimi was found to be 13 °C, as observed in the broth and the average MPD was ~6.96 log CFU/g, regardless of the storage temperature. Significant differences in LT values were observed in the sashimi sample and broth at all temperatures, but no significant differences in SGR values were observed between the sashimi sample and broth at 13 °C (*p* < 0.05). When MPD on flounder and salmon were compared to that on broth, they were significantly different at all temperatures (*p* < 0.05). In a comparison of growth kinetics on salmon sashimi, the LT values of *V. parahaemolyticus* on flounder were significantly shorter (*p* < 0.05), regardless of storage temperature. However, no significant differences in the values of SGR between flounder and salmon sashimi were observed at temperatures <24 °C (Table 3). Overall, the growth of *V. parahaemolyticus* in broth was more rapid compared to the growth on flounder and salmon sashimi (Table 3), indicating that the predicted growth model of *V. parahaemolyticus,* which were developed in broth, may not be appropriate for the risk assessment of *V. parahaemolyticus* in the sashimi sample. Moreover, more rapid growth of *V. parahaemolyticus* in flounder sashimi was observed compared to salmon sashimi. Our results demonstrate that behavior of *V. parahaemolyticus* may vary depending on the kind of fish (Figure 1). 

**Table 2:** Model for lag time (LT) and specific growth rate (SGR) of *V. parahaemolyticus* and *V. vulnificus* in broth, sashimi, and oyster.

Model	Parameter	Strain	R^2^	Equation
Broth	LT	*V. parahaemolyticus*	0.997	Y* = 19.31 + (−1227/T) + (20945/T^2^)
		*V. vulnificus*	0.997	Y = 2.784 + (−65.53/T) + (387.6/T^2^)
	SGR	*V. parahaemolyticus*	0.976	Y = {0.03489(T−4.213)}^2^
		*V. vulnificus*	0.996	Y = {0.03115(T + 1.316)}^2^
Floundersashimi	LT	*V. parahaemolyticus*	0.992	Y ^†^ = −28.04 + (−1037/T) + (−1.867/T^2^)
	SGR	*V. parahaemolyticus*	0.990	Y = {0.02017 (T−3.223)}^2^
Salmonsashimi	LT	*V. parahaemolyticus*	0.993	Y = −25.43 + (993.2/T) + (1792/T^2^)
	SGR	*V. parahaemolyticus*	0.939	Y = {0.01052(T + 13.52)}^2^
oyster	LT	*V. vulnificus*	0.950	Y = 24.80 + (−1211/T) + (17449/T^2^)
	SGR	*V. vulnificus*	0.930	Y = {0.01380 (T + 5.604)}^2^

* LT = a + (b/T) + (c/T^2^); **^†^** Y = {b(T–Tmin)}^2^.

**Table 3:** Effect of temperature on Lag time (LT) and specific growth rate (SGR) of *V. parahaemolyticus* in flounder and salmon sashimi and broth.

	Sample	13 °C	18 °C	24 °C	30 °C	36 °C
LT(h)	Flouder	^A ^51.35 ± 1.78 ^a^	^A ^30.96 ± 3.66 ^b^	^A ^14.79 ± 2.99 ^c^	^A ^3.90 ± 1.22 ^d^	^A ^2.77 ± 1.07 ^d^
	Salmon	^B ^61.16 ± 3.11 ^a^	^B ^36.81 ± 1.87 ^b^	^B ^18.54 ± 3.03 ^c^	^B ^6.98 ± 3.17 ^d^	^B ^5.64 ± 2.12 ^d^
	broth	^C ^48.64 ± 4.18 ^a^	^C ^16.94 ± 5.15 ^b^	^C ^2.52 ± 4.81 ^c^	^C ^2.22 ± 1.18 ^c^	^C ^1.61 ± 0.96 ^c,d^
SGR(log/h)	Flouder	^A ^0.037 ± 0.04 ^d^	^A ^0.105 ± 0.09 ^c^	^A ^0.152 ± 0.21 ^c^	^A ^0.304 ± 0.02 ^b^	^A ^0.435 ± 0.21 ^a^
	Salmon	^A ^0.059 ± 0.02 ^d^	^A ^0.103 ± 0.12 ^c^	^A ^0.185 ± 0.27 ^b^	^B ^0.219 ± 0.76 ^a,b^	^B ^0.256 ± 0.24 ^a^
	broth	^A ^0.035 ± 0.03 ^e^	^B ^0.160 ± 0.19 ^d^	^B ^0.561 ± 0.35 ^c^	^C ^0.847 ± 0.55 ^b^	^C ^1.184 ± 0.59 ^a^
MPD(log)	Flouder	^A ^6.53 ± 0.67 ^b^	^A ^7.30 ± 1.21 ^a^	^A ^7.21 ± 1.09 ^a^	^A ^7.60 ± 0.89 ^a^	^A ^7.46 ± 0.45 ^a^
	Salmon	^B ^5.54 ± 1.65 ^c^	^B ^6.51 ± 0.53 ^b^	^B ^6.45 ± 0.65 ^b^	^A ^7.34 ± 0.43 ^a^	^B ^6.95 ± 0.66 ^b^
	Broth	^C ^9.48 ± 0.87 ^a^	^C ^9.66 ± 1.07 ^a^	^C ^9.12 ± 1.19 ^a,b^	^B ^8.86 ± 0.53 ^b^	^C^ 9.74 ± 0.32 ^a^

Small letters mean (a, b, c, d) (n = 4) within a row with different superscripts are significantly different at the *p* < 0.05 level. Capital letters mean (A, B, C) (n = 4) within a column with different superscripts are significantly different at the *p* < 0.05 level.

Compared to the SGR values of pathogenic (0.42 log CFU/h) and nonpathogenic *V. parahaemolyticus* (0.42 log CFU/mL), a lower SGR value (0.30 log CFU/h) was also predicted for *V. parahaemolyticus* strain 33844. This indicates that care must be given when selecting a strain to develop a growth predictive model, and various strains isolated from seafood must be compared for their growth kinetics under various environmental conditions. These results again confirm the results regarding the study conducted by Burnham *et al.* [16], where strain to strain differences in the growth and survival of various *V. parahaemolyticus* and *V. vurnificus* were observed at refrigeration temperatures. 

The effect of temperature on the values of LT and SGR were determined and modeled (Table 2). The Davey equation for LT and the square root equation for SGR were used for the data collected in the broth. The minimum growth temperatures of *V. parahaemolyticus* and *V. vulnificus* in broth were 13 °C and 11 °C, respectively. In contrast to the results of this study, the minimum growth temperature of *V. parahemolyticus* in broth has been reported to be 5 °C and 8.3 °C [18]. Yoon *et al.* [19] also reported that pathogenic and nonpathogenic *V. parahaemolyticus* grew at 10 °C in broth. Burnham *et al.* [16] observed that five of eight *V. parahaemolyticus* strains and seven of eight *V. vulnificus* strains had increases in viable counts during 10 days of storage at 8 °C and 10 °C, respectively, with significant differences in growth between the various strains. 

The effect of temperature on LT and SGR in broth, sashimi, and oyster was also modeled (Table 2). The Davey equation was used in order to evaluate the LT of *V. parahaemolyticus*, respectively at 13, 18, 24, 30, and 36 °C, where growth was possible. A square root equation was used to determine the relationship between SGR and the storage temperature of *V. parahaemolyticus* on flounder, salmon, and broth in this work. In comparison to this study, Yang *et al.* [17] determined the growth and survival curves for a strain of pandemic *V. parahaemolyticus* Tgq + 01(serotype O3:K6) on salmon meat within the temperature range of 0 °C to 35 °C. They used salmon, which was soaked in sterile water containing 100 ppm chlorine. The minimum growth temperature of *V. parahaemolyticus* on salmon meat in their study was 12.1 °C, while the minimum growth temperature of *V. parahaemolyticus* on salmon sashimi was 13 °C in this study. At 30 °C, a 2-fold longer LT (6.98 h) was observed in our study compared to an LT value of 3.43 h in their study. Furthermore, the value of SGR for *V. parahaemolyticus* in salmon meat being twice as rapid compared to that of the *V. parahaemolyticus* in salmon sashimi (0.558 log CFU/h *vs.* 0.219 log CFU/h), indicated more rapid growth of *V. parahaemolyticus* Tgq+01(serotype O3:K6) on salmon meat than the *V. parahaemolyticus* ATCC strain in salmon sashimi. 

### 3.3. Growth Kinetics of Natural V. vulnificus in Shucked Oyster Meat

**Figure 2:**
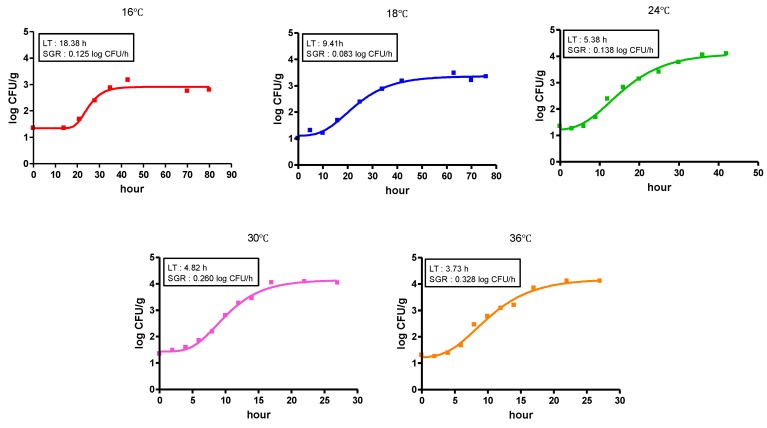
Growth curves of natural *V. vulnificus* in oyster as a function of time at various temperatures. Data (n = 4) were fitted using Equation (1). LT: lag time, SGR: specific growth rate symbols.

In the present study, we observed the growth of natural *V. vulnificus* in shucked oyster meat, which was purchased from a local grocery market. Since no growth of natural *V. vulnificus* was observed in oyster meat at a temperature lower than 16 °C, growth kinetics of naturally occurring *V. vulfnicius* in oyster meat was determined at 16, 18, 24, 30, and 36 °C (Figure 2). Recently, a predictive growth model for *V. parahaemolyticus* in Pacific oysters was developed [20], where 0.030 and 0.282 log_10_ CFU/h of growth rate was observed at 18.4 and 30.4 °C, respectively. On the other hand, the specific growth rate of *V. vulnificus* in oyster meat in our study was 0.080 log_10_ CFU/h at 18 °C and 0.328 log_10_ CFU/h at 30 °C (Figure 2). Although a direct comparison between their study and our study may not be appropriate, the growth of *V. vulnificus* was much faster than that of *V. parahaemolyticus* in oyster meat. In addition, DaSilva *et al.* [21] developed a predictive model for *V. vulnficus* in Eastern shellstock oysters (*Crassostrea Virginica*) over a temperature range of 5 to 30 °C. The estimated average growth rates at 15, 20, 25, and 30 °C were 0.022, 0.042, 0.087, and 0.093 log MPN/h, respectively. Overall, much lower growth rates of *V. vulnificus* were observed in shellstock oysters compared to our study, where we used shucked oyster meat. 

**Figure 3:**
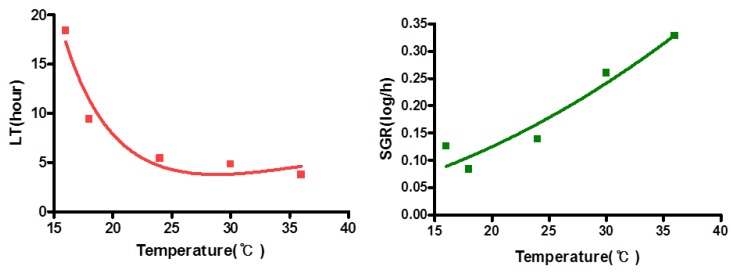
Effect of temperature on the values of lag time (LT) and specific growth rate (SGR) of natural *V. vulnificus* in oysters. Data (n = 4) for LT and SGR were fitted using Equations (2) and (3), respectively.

In addition, the minimum growth temperature of *V. vulnificus* in shellstock oysters was 15 °C in their work. In this study, the minimum growth temperature of natural *V. vulnificus* in oyster meat purchased from a local market was 16 °C (Figure 3), indicating that the growth of *V. vulnificus* may not be a major risk at retail markets as long as the shucked oyster pack is kept refrigerated. 

As shown in Table 4, the MPD of natural *V. vulnificus* in oysters reached a peak of ~4 log CFU/g from an initial contamination level of 1–1.3 log CFU/g, regardless of storage temperature, while MPD of the inoculated *V. vulnificus* strain 27562 in broth was over 9 log CFU/mL from an initial contamination level of 2.5 log CFU/mL. 

**Table 4:** Comparisons of maximum population density (MPD) of natural *V. vulnificus* in oyster and inoculated *V. vulnificus* (ATCC 27562) in broth.

Sample		16 °C	18 °C	24 °C	30 °C	36 °C
Broth	Initial density *	-	2.44 ± 0.91	3.33 ± 0.44	3.32 ± 0.29	2.75 ± 0.45
	MPD	-	8.70 ± 1.09	9.10 ± 0.45	8.90 ± 0.34	9.00 ± 0.89
Oyster	Initial density ^†^	1.34 ± 0.22	1.00 ± 0.32	1.31 ± 0.46	1.30 ± 0.23	1.34 ± 0.17
	MPD	2.79 ± 0.58	3.47 ± 0.12	4.13 ± 0.54	4.08 ± 0.52	4.10 ± 0.36

* Initial inoculation level; **^†^** Initial contamination level (n = 4) in oyster.

These results indicate that the populations of naturally occurring *V. vulnificus* in oysters did not reach the maximum population density observed in the broth. However, the infectious dose of *V. vulnifcus* for the high risk group is 2 log CFU/g, therefore, for the protection of consumers careful storage and consumption guidelines for oysters at retail markets and restaurants must be emphasized. In this study, only *V. vulnifius* was consistently detected in oysters during the period of September to March and the average population of *V. vulnifius* in shucked oyster meat at the retail grocery market was 1–1.3 log CFU/g.

Lee *et al.* [24] also reported *V. parahaemolyticus* as the dominant *Vibrio* in retail oysters during August to September. In their study, the total *Vibrio* number was ~4 log MPN/g and the average number of *V. parahaemolyticus* in Korean retail oysters was ~3 log MPN/g. This discrepancy might between these two studies be due to the seasonal differences during the sampling period as well as the different purchase sites of the oyster samples. 

**Figure 4:**
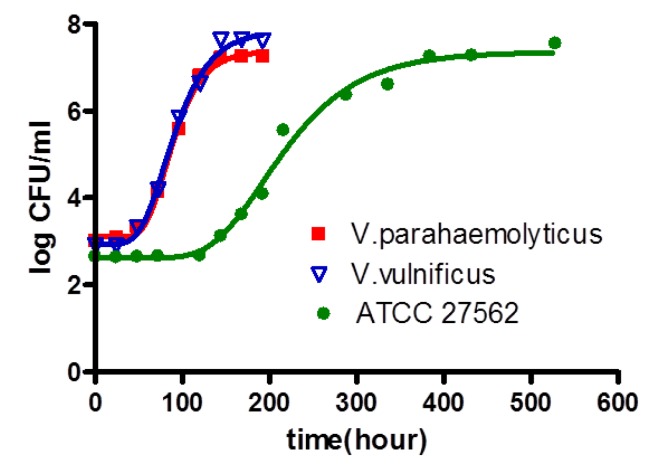
Comparison the growth curves of inoculated *V. vulnificus* and *V. parahaemolyticus* isolated from oyster and *V. vulnificus* ATCC 27562 in broth at 11 °C. Data (n = 4) were fitted using Equation (1).

Although it was not consistent, we were able to isolate both *V. parahaemolyticus* and *V. vulnificus* from shucked oyster meat purchased from a grocery market in Korea. Thus, these organisms were inoculated into broth and stored at 11 °C (Figure 4), which was the minimum growth temperature of natural *V. parahaemolyticus and V. vulnificus* in broth. As previously mentioned, the minimum growth temperature for strains *V. parahaemolyticus* 33844 and *V. vulnificus* 27562 purchased from ATCC were 13 °C and 11 °C in broth, respectively (Table 1). Again, this result indicates that the behaviors of naturally occurring *Vibrio* strains in oysters are different from the behavior of the reference ATCC strain. As shown in Figure 4, *V. vulnificus* ATCC strain 27562 grew more slowly than *V. parahaemolyticus* and *V. vulnificus* isolated from retail oysters, indicating that growth models of *Vibrio.* spp*.* in the literature should be validated with natural *V. parahaemolyticus* and *V. vulnificus* in oysters. 

## 4. Conclusions

*V. vulnificus* was more frequently detected than *V. parahaemolyticus* in oysters from grocery markets in Korea from September to March. In this study, natural *V. vulnificus* in oysters grew to ~4 log CFU/g, regardless of the storage temperature tested. However, when *V. vulnificus* strain 27562 and naturally occurring *V. vulnificus* isolated from retail oysters were inoculated in broth, they grew to ~9 log CFU/mL in broth, regardless of storage temperature. These results indicate that the populations of *V. vulnificus* in oysters did not reach the maximum population density observed in the broth. In addition, natural *V. parahaemolyticus * and *V. vulnificus* were isolated from retail oysters and their growth curves were compared to those of *V. vulnificus* ATCC strain 27562. Their minimum growth temperature was 11 °C in broth, while the minimum growth temperature regarding *V. parahaemolyticus* strain 33844 was 13 °C in broth and sashimi. These results indicate that storage of ready to eat sashimi and oysters at temperatures < 10 °C at retail markets will control the growth of *Vibrio* spp. and thus minimize the risk of illness associated with *Vibrios* due to raw and ready-to-eat seafood consumption. Therefore, strict temperature guidelines during the transportation and selling of raw and ready-to-eat seafood must be implemented at retail markets. The present data will be used to develop the tertiary growth model for *V. parahaemolyticus* and *V. vulnificus* from harvest to the point of sale in order to manage the risk of illness as a result of sashimi and raw oyster consumption. However, more extensive research for evaluating the growth behaviors of *V. parahaemolyticus* and *V. vulnificus* strains in various seafoods is needed for an overall risk assessment of raw seafood consumption. 
