# *Correction*: Kim, Y.W.; Lee, S.H.; Hwang, I.G.; Yoon, K.S. Effect of Temperature on Growth of *Vibrio parahaemolyticus* and* Vibrio vulnificus* in Flounder, Salmon Sashimi and Oyster Meat. *Int. J. Environ. Res. Public Health* 2012, *12*, 4662-4675

**DOI:** 10.3390/ijerph10073014

**Published:** 2013-07-18

**Authors:** Yoo Won Kim, Soon Ho Lee, In Gun Hwang, Ki Sun Yoon

**Affiliations:** 1 Department of Food and Nutrition, Hoeki-dong Dongdaemun-Ku, Kyung Hee University, Seoul 130-701, Korea; E-Mail: ryuwon@gmail.com; 2 Food Microbiology Division, National Institute of Food and Drug Safety Evaluation, Korea Food and Drug Administration, 363-700, Korea; E-Mails: leesh13@kfda.go.kr (S.H.L.); hwangig@kfda.go.kr (I.G.H)

The authors wish to add the following correction on their paper published in IJERPH [1], doi: 10.3390/ijerph9124662, website: http://www.mdpi.com/1660-4601/9/12/4662. “*Vibrio paraphemolyticus*” in the article title should be “*Vibrio parahaemolyticus*”.

The authors apologize for the inconvenience.
